# Cytotoxic clinical isolates of *Pseudomonas aeruginosa* identified during the Steroids for Corneal Ulcers Trial show elevated resistance to fluoroquinolones

**DOI:** 10.1186/1471-2415-14-54

**Published:** 2014-04-24

**Authors:** Durga S Borkar, Nisha R Acharya, Chelsia Leong, Prajna Lalitha, Muthiah Srinivasan, Catherine E Oldenburg, Vicky Cevallos, Thomas M Lietman, David J Evans, Suzanne M J Fleiszig

**Affiliations:** 1 Department of Ophthalmology, University of California, 94143 San Francisco, CA, USA; 2 Francis I. Proctor Foundation, University of California, 94143 San Francisco, CA, USA; 3 School of Optometry, University of California, 688 Minor Hall, 94720 Berkeley, CA, USA; 4 Aravind Eye Care System, Madurai, Tamil Nadu, India; 5 Department of Epidemiology and Biostatistics, University of California, 94143 San Francisco, CA, USA; 6 College of Pharmacy, Touro University California, 94592 Vallejo, CA, USA; 7 Programs in Vision Science, Infectious Diseases and Immunity, and Microbiology, University of California, 94720 Berkeley, CA, USA

**Keywords:** *P. aeruginosa*, Microbial keratitis, SCUT, Fluoroquinolone resistance, ExoU

## Abstract

**Background:**

To determine the relationship between type three secretion genotype and fluoroquinolone resistance for *P. aeruginosa* strains isolated from microbial keratitis during the Steroids for Corneal Ulcers Trial (SCUT) and for two laboratory strains, PA103 and PAO1.

**Methods:**

Confirmed *P. aeruginosa* isolates from the SCUT were divided into *exoU*(+) or *exoU*(−). The *exoU*(+) strains contained the gene encoding ExoU, a powerful phospholipase toxin delivered into host cells by the type three secretion system. Isolates were then assessed for susceptibility to fluoroquinolone, cephalosporin, and aminoglycoside antibiotics using disk diffusion assays. Etest was used to determine the MIC of moxifloxacin and other fluoroquinolones. Laboratory isolates in which the *exoU* gene was added or deleted were also tested.

**Results:**

A significantly higher proportion of *exoU*(+) strains were resistant to ciprofloxacin (p = 0.001), gatifloxacin (p = 0.003), and ofloxacin (p = 0.002) compared to *exoU*(−) isolates. There was no significant difference between *exoU*(+) or *exoU*(−) negative isolates with respect to susceptibility to other antibiotics except gentamicin. Infections involving resistant *exoU*(+) strains trended towards worse clinical outcome. Deletion or acquisition of *exoU* in laboratory isolates did not affect fluoroquinolone susceptibility.

**Conclusions:**

Fluoroquinolone susceptibility of *P. aeruginosa* isolated from the SCUT is consistent with previous studies showing elevated resistance involving *exoU* encoding (cytotoxic) strains, and suggest worse clinical outcome from infections involving resistant isolates. Determination of *exoU* expression in clinical isolates of *P. aeruginosa* may be helpful in directing clinical management of patients with microbial keratitis.

## Background

*Pseudomonas aeruginosa* is a leading cause of bacterial keratitis involving contact lens wear or ocular injury [1-5]. While the pathogenesis of *P. aeruginosa* keratitis is complex, studies with in vivo models have shown that the type three secretion system (T3SS) is a significant contributor to disease pathogenesis [6-8]. Similar results have been obtained in murine models of acute pneumonia [9], and expression of the T3SS is associated with worse patient outcomes in ventilator-associated pneumonia and bacteremia [10,11]. Other clinical studies have also shown poor patient outcomes from *P. aeruginosa* infections are also associated with antimicrobial drug resistance [12,13], a long recognized trait of this opportunistic pathogen [14].

The T3SS effector ExoU is a potent phospholipase that is cytotoxic towards host cells [15,16]. Several studies have shown that the *exoU* gene is selected for in clinical isolates from microbial keratitis [17-19], especially in contact lens-associated cases [20]. Interestingly, several other studies have also shown a relationship between *exoU* expression and resistance of *P. aeruginosa* to contact lens disinfectants [21], and to multiple antimicrobials [22], especially fluoroquinolones [23]. In the latter instance, it was shown that *P. aeruginosa* clinical isolates encoding *exoU* were more likely to encode multiple mutations in quinolone target genes, e.g. *gyrA* and *parC*, and suggested a co-evolution of these traits [24].

The Steroids for Corneal Ulcers Trial (SCUT), was a randomized controlled trial investigating the effects of adjunctive corticosteroid therapy to fluoroquinolone (moxifloxacin) treatment on the outcomes of bacterial keratitis [25,26]. Analysis of moxifloxacin resistance of bacterial keratitis isolates (including *P*, *aeruginosa*) from the SCUT study showed that increased resistance to this fluoroquinolone in vitro (i.e. increased minimum inhibitory concentration) was associated with poorer clinical outcomes, including reduced visual acuity at 3 weeks, a larger ulcer, and slower corneal re-epithelialization [27]. Here, we analyzed *P. aeruginosa* strains isolated from the SCUT study, and two laboratory isolates and their T3SS mutants, for their in vitro susceptibility to fluoroquinolones and other antimicrobials including cephalosporins and aminoglycosides. Antimicrobial classes and individual agents tested were chosen because of their common clinical empirical use against bacterial keratitis [28]. The objective was to determine if the SCUT clinical isolates and laboratory strains of *P. aeruginosa* also showed a relationship between fluoroquinolone resistance and *exoU* expression.

## Methods

### a) Clinical and laboratory isolates of P. aeruginosa and T3SS mutants

The *P. aeruginosa* isolates used were obtained from the Steroids for Corneal Ulcers Trial (SCUT), a randomized controlled trial investigating the effect of adjunctive corticosteroids on outcomes in bacterial keratitis [25]. The SCUT study was conducted with the approval of the University of California, San Francisco; Aravind Eye Hospital, Madurai, India; Dartmouth-Hitchcock Medical Center, Hanover, NH. Informed consent was obtained from all study participants. Corneal isolates were collected from patients with culture-confirmed bacterial keratitis. The isolates used in the present study were previously confirmed as *P. aeruginosa* using growth morphology, Gram stain, and API test strips, and their type three secretion gene profile was determined [29]. Presence of *exoU* was determined by polymerase chain reaction using primers and conditions described in detail by Ledbetter *et al*. [30]. Two wild-type laboratory strains of *P. aeruginosa* (PA103, encodes *exoU*) and PAO1 (does not encode *exoU*) were tested. For strain PA103, two T3SS mutants PA103*exsA*::Ω and PA103Δ*exoU* were also used. Strain PAO1 was complemented with a plasmid encoding *exoU* (pUCP*exoU*) or a plasmid control (pUCP18).

### b) Antibiotic susceptibility testing

Susceptibility to moxifloxacin, the fluoroquinolone antibiotic used in the SCUT, was measured using the Etest method (AB BIODISK, Solna, Sweden) for all isolates using a standardized inoculum of 1 × 10^8^ CFU/mL on Mueller-Hinton agar plates, which were then incubated at 35°C. Disc diffusion susceptibility testing was performed for various other antibiotics (other fluoroquinolones, cephalosporins, and aminoglycosides) and *P. aeruginosa* strains classified as susceptible, intermediate, or resistant to each antibiotic tested based on the Clinical and Laboratory Standards Institute (CLSI) guidelines [31]. Since CLSI guidelines do not provide susceptibility ranges for the MIC of moxifloxacin for *P. aeruginosa*, susceptibility ranges for ciprofloxacin were used for Etest results. Laboratory personnel were masked as to *P. aeruginosa* strain identity. Laboratory isolates were tested after the conclusion of the SCUT using similar methods, which have been described previously [25]. An ATCC *P. aeruginosa* strain (ATCC 27853) was used for quality control when performing antibiotic susceptibility testing for laboratory strains.

### c) Visual acuity

SCUT patients with culture-confirmed bacterial keratitis were evaluated at multiple time points, including enrollment and three months, by certified refractionists who performed visual acuity examinations. Visual acuity was measured as best spectacle-corrected visual acuity (BSCVA) in logMAR units, with 0.1 logMAR corresponding to approximately one line of acuity. Laboratory personnel were masked to the clinical data.

### d) Statistical analysis

Differences in disc diffusion susceptibility for various antibiotics, and mean minimum inhibitory concentration (MIC) of moxifloxacin, between *exoU*(+) or *exoU*(−) strain types were compared using Fisher’s exact test and the Student’s t-Test, respectively. The t-Test was also used to compare the change in BSCVA between enrollment and three months between patients with corneal ulcers caused by resistant or non-resistant *exoU*(+) *P. aeruginosa*.

## Results

Of 500 patients enrolled in the SCUT, 110 had a corneal ulcer involving *P. aeruginosa*[32]. Confirmed *P. aeruginosa* isolates that were available for study were divided into two groups according to presence or absence of the *exoU* gene. Table 1 shows disc diffusion antimicrobial susceptibility data for 19 *exoU*(+) and 75 *exoU*(−) isolates. A significantly higher proportion of *exoU*(+) strains were resistant to ciprofloxacin (p = 0.001), gatifloxacin (p = 0.003), and ofloxacin (p = 0.002) compared to *exoU*(−) isolates. There was no significant difference between *exoU*(+) or *exoU*(−) negative isolates with respect to susceptibility to the cephalosporins tested, or to the aminoglycosides amikacin and tobramycin. Interestingly, there were significantly more gentamicin resistant *exoU*(+) isolates (p = 0.02).

**Table 1:** **Antibiotic susceptibility**^**1 **^**of SCUT Isolates of *P*. *aeruginosa *** **and T3SS genotype**

	***exoU*** **(+) (n = 19)**		***exoU*** **(-) (n = 75)**		***P*-value**^**2**^
	**Resistant**	**Non-resistant**	**Resistant**	**Non-resistant**	
*Fluoroquinolones*					
Ciprofloxacin^3^	5 (28%)	13 (72%)	1 (1%)	74 (99%)	0.001
Gatifloxacin	5 (26%)	14 (74%)	2 (3%)	73 (97%)	0.003
Ofloxacin	6 (32%)	13 (68%)	3 (4%)	72 (96%)	0.002
*Cephalosporins*					
Cefotaxime	3 (16%)	16 (84%)	9 (12%)	66 (88%)	0.70
Ceftazidime	3 (16%)	16 (84%)	5 (7%)	70 (93%)	0.20
*Aminoglycosides*					
Amikacin	1 (5%)	18 (95%)	1 (1%)	74 (99%)	0.37
Gentamicin	5 (26%)	14 (74%)	4 (5%)	71 (95%)	0.02
Tobramycin	3 (16%)	16 (84%)	2 (3%)	73 (97%)	0.054

^1^Disc diffusion assay.

^2^Fisher’s exact test.

^3^Disc diffusion susceptibility data for ciprofloxacin not available for one *exoU*(+) isolate (n = 18).

Of the 19 *exoU*(+) corneal isolates, 4 were resistant to gatifloxacin, ciprofloxacin, and ofloxacin based on disc diffusion susceptibility testing, and had an MIC greater than or equal to 4 micrograms/ml. The BSCVA of patients with ulcers caused by the resistant ExoU(+) isolates improved, on average, approximately six lines less from enrollment compared to patients with ulcers caused by non-resistant ExoU(+) isolates (−0.38 logMAR vs. -1.04 logMAR) although this difference was not statistically significant (p = 0.08).

**Figure 1:**
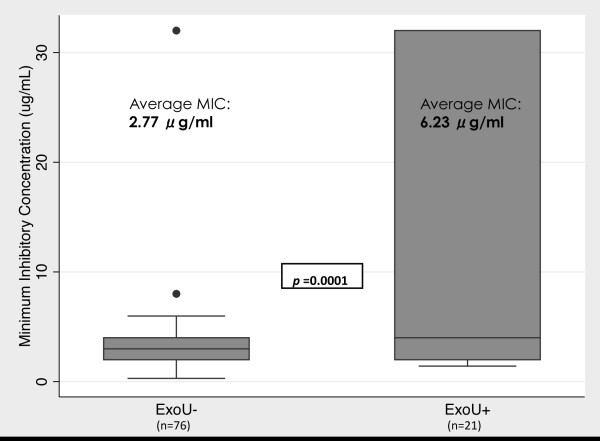
**Minimum inhibitory concentration (MIC) of moxifloxacin for *P*. *aeruginosa *clinical isolates with or without the *exoU *gene.** The median for each group is represented by the line in the middle of each box. The interquartile range (IQR), the span of the 25^th^ to 75^th^ percentiles, is denoted by the lower and upper bounds of each box, respectively. The whiskers extend from the smallest MIC within 1.5 × IQR below the 25^th^ percentile to the largest MIC within 1.5 × IQR above the 75^th^ percentile. Individual data points denote values outside this range. Note that 3 additional isolates were identified and included compared to disk diffusion assay data; 2 were *exoU*(+) and 1 was *exoU*(−). The *P*-value displayed was obtained by two-group mean-comparison t-Test of the log, in base 2, of the MIC. The average MIC shown for each subgroup is the mean of the log, in base 2, of the MIC transformed to MIC in micrograms/ml.

In another set of experiments, the MIC of moxifloxacin was determined and compared for 21 *exoU*(+) and 76 *exoU*(−) SCUT isolates of *P. aeruginosa*. Figure 1 shows that isolates encoding *exoU* had, on average, a greater than two-fold higher MIC compared to *exoU*(−) strains (p = 0.0001).

Laboratory isolates of *P. aeruginosa* were also tested to determine if genetic deletion of the T3SS, or deletion or acquisition of *exoU*, affected bacterial susceptibility to fluoroquinolones or other antibiotics using disc diffusion and Etest assays (Table 2). Neither deletion of the T3SS or *exoU* in the cytotoxic strain PA103, nor acquisition of *exoU* in the invasive strain PAO1, affected sensitivity to any antimicrobial tested.

**Table 2:** **Antibiotic susceptibility testing results for *P*. *aeruginosa *** **laboratory isolates**

	**Disc diffusion susceptibility**^**1**^							**Etest**^**2**^
**Strain**	**Cipro**	**Gati**	**Oflox**	**Ceftaz**	**Gent**	**Tob**	**Moxi**	**Moxi**
PA103	S	S	S	S	S	S	S	S (1.0)
PA103 *exsA*::Ω	S	S	S	S	S	S	S	S (0.5)
PA103 Δ*exoU*	S	S	S	S	S	S	S	S (0.5)
PAO1	S	S	S	S	S	S	S	S (0.5)
PAO1 + pUCP *exoU*	S	S	S	S	S	S	S	S (0.5)
PAO1 + pUCP18	S	S	S	S	S	S	S	S (1.0)
ATCC 27853 (QC)	S	S	S	S	S	S	I	I (2.0)

^1^S = Sensitive; I = Intermediate; R = Resistant. Ranges used to determine susceptibility were taken from the Clinical and Laboratory Standards Institute guidelines. QC = Quality Control.

^2^S = Sensitive; I = Intermediate; R = Resistant. Numbers in parentheses represent the MIC (micrograms/ml).

## Discussion

Topical fluoroquinolones are commonly used in the treatment of bacterial keratitis, in which *P. aeruginosa* is a leading causative pathogen. The data presented in this study show the antimicrobial resistance patterns of *P. aeruginosa* strains (94) isolated from patients during the SCUT. The data show a significant increase in the number of isolates resistant to fluoroquinolones if the bacteria encode the type three secreted cytotoxin ExoU. The data also confirm that deletion or acquisition of *exoU* gene does not affect fluoroquinolone susceptibility. These data are consistent with previous studies showing an association of *exoU* expression with fluoroquinolone resistance in *P. aeruginosa*, and support the hypothesis that T3SS expression and fluoroquinolone resistance are co-selected traits in both ocular and non-ocular clinical isolates [22,23]. Indeed, prior studies have found a significantly higher rate of *gyrA* mutations, combined with mutations in other fluoroquinolone target genes e.g. *parC*, in *exoU* encoding *P. aeruginosa* isolates [23,24].

It was of interest that we also found significantly more gentamicin resistant *exoU*(+) isolates in the study, although this did not occur for other aminoglycosides. This finding is consistent with a previous study looking at bacteremia isolates of *P. aeruginosa* in which the presence of *exoU* was also associated with increased resistance to gentamicin, but not amikacin [11]. It would be of interest to determine if gentamicin resistance in these ocular *exoU*(+) isolates relates to specific mutations in drug target genes, as shown for fluoroquinolones [24].

In bacterial keratitis, antibiotic susceptibility likely plays a role in clinical outcome, suggesting a role for testing antibiotic susceptibility in management decisions [27,33]. Interestingly, we found that SCUT patients with ulcers caused by fluoroquinolone resistant *exoU*(+) *P. aeruginosa* isolates trended toward worse visual outcomes at three months compared to those with ulcers caused by non-resistant *exoU*(+) isolates. These results could reflect increased bacterial persistence in the cornea, allowing for greater tissue damage from bacterial toxins (including ExoU), and from host immune infiltrative responses.

The continuing emergence of antimicrobial resistant strains has the potential to influence “optimal” treatment of *P. aeruginosa* keratitis. However, *P. aeruginosa* resistance rates to fluoroquinolones and aminoglycosides, common choices for management of this condition, remain relatively low and stable in many places, although they can vary widely by geographical location and time [22,34-36]. Our study, and those of others [11,22-24], however, suggest that the combination of *exoU* and antibiotic resistance may be particularly deleterious to the patient and rapid identification of these isolates, if possible, could help optimize treatment.

## Conclusion

Fluoroquinolone susceptibility of *P. aeruginosa* isolated from the SCUT is consistent with previous studies showing elevated resistance involving *exoU* encoding (cytotoxic) strains, and suggest worse clinical outcome from infections involving these resistant isolates. Increased gentamicin resistance is also associated with *exoU*(+) strains. Determination of *exoU* expression in clinical isolates of *P. aeruginosa*, and antibiotic susceptibility testing, may be helpful in directing optimal clinical management of patients with microbial keratitis if methods could be developed with sufficient speed and accuracy, and which could be applied in a clinical setting.

## Competing interests

Dr. Suzanne Fleiszig is a paid consultant for Allergan, Irvine, CA. That work is unrelated to the content of this manuscript. There are no competing interests for any of the authors.

## Authors’ contributions

Study design and analysis: DB, SF, NA, DE, PL, MS, TL. Experiments: DB, CL, CO, VC. Manuscript writing: DB, DE, NA, SF. Supervision: SF, NA, TL. All authors read and approved the final manuscript.

## Pre-publication history

The pre-publication history for this paper can be accessed here:

http://www.biomedcentral.com/1471-2415/14/54/prepub
